# Fractional Q-Switched 1064 nm Laser for Treatment of Atrophic Scars in Asian Skin

**DOI:** 10.3390/medicina58091190

**Published:** 2022-09-01

**Authors:** Steven Paul Nisticò, Mario Sannino, Gaia Fasano, Miriam Marigliano, Francesca Negosanti, Luigi Bennardo, Giovanni Cannarozzo

**Affiliations:** 1 Department of Health Sciences, Magna Graecia University, 88100 Catanzaro, Italy; 2 Villa Bella Dermatologic Center, 40121 Bologna, Italy; 3 Unit of Lasers in Dermatology, Tor Vergata University, 00100 Rome, Italy

**Keywords:** Nd: YAG laser, fractional Q-switched, scars, Asian skin

## Abstract

*Background and Objectives:* Asian patients with Fitzpatrick skin type III–IV are a less studied subtype of patients in the medical literature. Q-Switched, 1064 nm neodymium-doped yttrium aluminum garnet (Nd: YAG) laser with a fractionated beam profile (QSF) is a new modality that was reported to be effective in the treatment of scars. This study aims to evaluate the efficacy and safety of QSF Nd: YAG laser in treating scars in Asian patients. *Materials and Methods:* A total of 29 Subjects were treated with 1064 nm QSF laser. Each patients had three treatments with a fractionated microlens array handpiece every 8 weeks). Efficacy of treatment was evaluated using the Goodman and Baron’s quantitative grading scale before and 3 months after the last treatment. *Results:* All 29 patients treated had significant improvement of acne scars according to Goodman and Baron’s Quantitative Global Acne Scarring Grading System. No side effect has been observed except some minor erythematous reactions in three patients. *Conclusions:* Our results confirm that the 1064 nm QSF Nd: YAG laser is a safe and effective technique for treating scars in Asians.

## 1. Introduction

Scars result from abnormal wound healing. Genetic, systemic, and local factors, such as inflammatory and proliferative processes, lead to excessive extracellular matrix deposition and overgrowth scars formation. The most common scar types are atrophic, hypertrophic scars, keloids, and striae distensae [1].

Acne is an inflammatory disorder that causes atrophic scars. Many therapeutic strategies can be used for atrophic scars, such as hyaluronic acid fillers, chemical peeling, and lasers [2].

Several treatments have been proposed for hypertrophic and keloid scars, like intralesional steroid injection, steroid tapes, surgical revision, cryotherapy, radiotherapy, and laser therapy [3]. Treatment should also consider skin phototype, anatomic site, and racial differences. White and Asian skin presents many anatomic and physiological differences that lead to different kinds of side effects. Acne, hypertrophic, and keloid scars are more common in Asian patients than Caucasians [4]. Post-inflammatory hyperpigmentation (PIH) and keloids are the most common adverse events after laser treatment in Asian skin and force operators to use conservative parameters [5].

In the past decade, the safety and efficacy of a Q-Switched domain, 1064 nm neodymium-doped yttrium aluminum garnet (Nd: YAG) laser have been widely demonstrated in many different conditions like melasma, tattoo removal, hair removal, and skin rejuvenation [6,7,8,9,10]. A growing interest in treating scars with fractional Nd: YAG laser is present [11].

To the best of our knowledge, QSF 1064 nm laser has never been used to treat scars in Asian patients, and this study aims to investigate the effectiveness and safety of this laser.

## 2. Materials and Methods

The study retrospectively recruited patients at the Magna Graecia University of Catanzaro, La Sapienza University of Rome, and Villa Bella Dermatological Centre in Bologna.

Twenty-nine Asian subjects (2 males and 27 females), with Fitzpatrick skin type III-IV and residual acne scars evaluated by Goodman and Baron’s Quantitative Global Acne Scarring Grading System, were assessed by an investigator.

Inclusion criteria were as follows: age ≥ 18 years, presence of cosmetic scars, no severe underlying pathologies, and compliance to follow-up.

Exclusion criteria included a history or suspicion of active infection at the sites of treatment, immunocompromised status, steroid and immunosuppressive drug assumption, history of skin cancer, recurrent herpes viral infection, chronic diseases influencing the skin (e.g., diabetes, autoimmune disease), oral retinoid within six months before the treatment, light hypersensitivity, taking phototoxic medication (such as some antibiotics), history of chemical peeling one month prior to the study, facial laser treatment in the past three months, history of connective tissue disease, pregnancy, and breastfeeding status.

All patients provided informed written consent.

### 2.1. Laser Device

QFS 1064 nm Nd: YAG laser (Smart PICO^®^; DEKA M.E.L.A. S.p.A., Calenzano, Italy) was used for all laser treatments. SmartPICO^®^ has multiple handpieces with several different spot sizes for all the targets. Each spot has a microlens array (MLA), which optimizes energy delivery and fractionates the laser beam into microbeams with circular diameter.

The fractionated system produces micro-injuries in micro-regions that trigger the new dermal collagen formation and a repair process.

### 2.2. Laser Session Protocol

Patients were treated by the same dermatologist with a 1064 nm QSF laser (Spot size of 9 mm fractionated handpiece for scar treatment and 0.7–0.8 J/cm^2^ of fluence; repetition rate, 5 Hz; pulse width, 450 picoseconds. Each patient had 3 treatments with a fractionated microlens array handpiece every 8 weeks. No topical anesthesia was used prior to treatment.

Desired endpoints were immediate moderate erythema and mild oozing of bloody serous exudates, a sign of epidermal ablation. A dynamic cooling device during the treatment was used; the technique results in the reduction of skin temperature to 5 °C and −9 °C to approximately 200 μm of superficial tissue. Therefore, we safely used high fluences with a good margin of safety.

After the treatment, patients were instructed to use an antibiotic cream twice a day for 7–10 days to avoid sun exposure and sun protection SPF50+ over the treated area for 30 days. All subjects received treatment every eight weeks for three sessions and followed up 3 months after the last procedure.

### 2.3. Efficacy and Safety

Before and after each session, the patient was visited and photographed with a professional dermatological camera with and without polarized light (Anthology—DEKA M.E.L.A., Florence, Italy) at different angles (frontal and profile from right and left side).

Efficacy was evaluated at baseline, and 3 months after procedures.

Efficacy of the treatment was assessed using Goodman and Baron’s quantitative grading scale assessed by an investigator as compared with the baseline digital photographs before and 3 months after the last treatment (Table 1) [12]. Also, a Visual Analogue Scale (VAS) of 10 points (0, none; 1–2, slight pain; 3–6, moderate pain; 7–8, severe pain, 9–10, intolerable pain) to evaluate pain was used to evaluate patients’ tolerance.

Efficacy of the treatment was assessed using the multispectral analysis which is an optical imaging method to characterize skin tissue with high resolution and discrimination the changes in the macroscopic structure. The spectral imaging device perform quantitative per-pixel spectral analysis of tissue for evaluation of laser treatment of skin lesions.

The appearance of side effects such as blistering, scarring, burns, hypopigmentation, or hyperpigmentation was also monitored.

Statistical analysis was executed using a paired Student’s *t* test. Statistica 14.0 was used to analyze data (mean, standard deviations, and rate calculations) (TIBCO Software, Palo Alto, CA, USA).

## 3. Results

**Figure 1:**
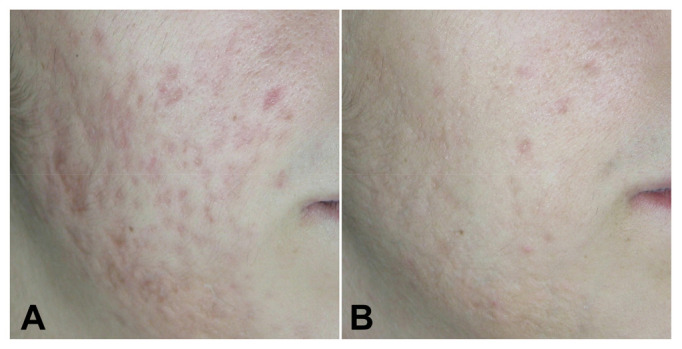
Patient 14 before (**A**) and 3 months after (**B**) treatments.

**Figure 2:**
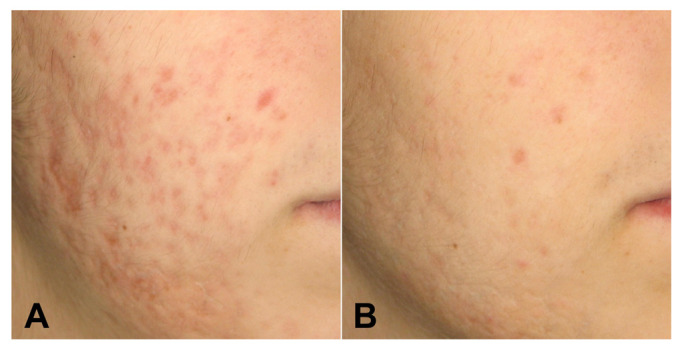
Patient 14 before (**A**) and 3 months after (**B**) treatments under polarized light.

**Figure 3:**
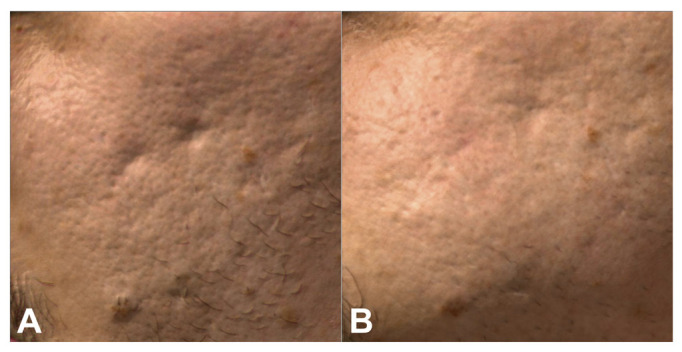
Patient 14 before (**A**) and 3 months after (**B**) treatments at multispectral analysis.

All the patients completed the study. The cohort was a total of 29 patients (93% female and 7% male) with a mean age of 29.66 ± 7.77 years. Participants’ skin color ranged (using the Fitzpatrick scale) from type II (n = 1 3.4%) to type III (n = 14; 48.3%) and type IV (n = 14; 48.3%). All treated patients had significant improvement of acne scars according to Goodman and Baron’s Quantitative Global Acne Scarring Grading System and photographic evaluation. The scores decreased significantly from baseline to 3 months follow-up after the last treatment (initial score 22.59 ± 7.44; final score 14.14 ± 7.06; *p* < 0.001). Treatment was well tolerated with a low pain VAS score (2.45 ± 1.09) (Figure 1, Figure 2 and Figure 3).

The most common immediate post-treatment side effects were erythema, edema, and exfoliation that resolved within 10 days. No prolonged erythema and no pigmentation alterations were reported at 12 weeks after treatment. No severe adverse events were observed. Post-inflammatory hyperpigmentation was not observed in our study. Patients’ characteristics are reported in Table 2.

## 4. Discussion

Scars are fibroproliferative disorders resulting from an alteration of wound healing processes; can be distinguished into atrophic scars, hypertrophic scars, keloids, and striae distensae [13].

Atrophic scars result from collagen fibers and subcutaneous fat loss during an inflammatory process such as acne vulgaris; hypertrophic scars are raised within the injury site and develop within 4–8 weeks after the injury; keloids, instead, extend beyond the site of injury [14]. Many traumas can lead to scarring, such as surgery, burns, piercings, infections, insect bites, and physical trauma [15]. Patients reported functional and cosmetic problems such as sensation of tension, itching, and discomfort that affected their quality of life [16].

According to the width and depth, atrophic acne scars can be classified into icepick, rolling, and boxcar scars; classification helps us choose the best treatment for each patient. Icepick scars extend vertically into the dermis and subcutaneous tissue and cannot be treated with superficial resurfacing; rolling scars are shallower than icepick scars and result from abnormal fibrous anchoring; boxcar scars, instead, are rectangular and vertical depressions; the shallow ones can be treated with superficial skin resurfacing [17].

Lasers are widely used for acne scars treatment; ablative and non-ablative are valid treatment options and can be used in fractional modality. Fractional laser resurfacing cause microscopic columns of epidermal and dermal tissue damage and is a good option for rolling and boxcar scars [18].

Ablative lasers, 10,600-nm CO_2_ and 2900-nm Er: YAG, target water in the skin, causing epidermal and dermal destruction and stimulating the formation of new collagen [19]. Prolonged erythema, PIH, and long recovery time have made ablative lasers less popular [20]. Non-ablative lasers, 1064-nm Nd: YAG and 585-nm pulsed dye laser (PDL), target the dermis and leave the epidermis intact, so they can be considered less invasive than ablative lasers, and, furthermore, aim to stimulate collagen and dermal remodeling [21,22,23,24]. Shallow boxcar and rolling scars, compared to icepick scars, have a more significant improvement [25]. Non-ablative fractional lasers (NAFL) have a faster recovery time and fewer adverse events than fractional ablative lasers (AFL). However, more sessions are required, and improvement is not as much as with AFL [26].

Different treatment modalities are used for hypertrophic, keloid scars, and striae distensae. For atrophic scars, other possible treatments are dermabrasion, micro-needling, platelet-rich plasma, and radiofrequency [27]. The goal of current strategies is to reduce inflammation with a different mechanism. Intralesional steroid injections, steroid tapes, and steroid ointments reduce inflammation and decrease the proliferation of fibroblast and collagen synthesis, but the most common side effects are atrophy and telangiectasia [28]. Surgical revision or excision of scars is a traditional treatment that aims to reduce skin tension and can be carried out at least one year after scar formation [29]. Results are often not encouraging due to the recurrence rate, especially for keloids [29]. Combination therapy with postoperative steroid application can improve surgical outcomes [30]. Cryotherapy is used to induce vascular damage and scar tissue necrosis and can be associated with intralesional steroid injections to induce a higher success rate [31].

Several laser treatments with different wavelengths are primarily reported, including 585-nm PDL, 1064-nm Nd: YAG laser, 308-nm excimer laser, non-ablative 1450-nm diode laser, ablative fractional CO_2_ resurfacing, and fractional Er: YAG laser [32,33]. PDL 585-nm and Nd: YAG 1064-nm lasers are frequently used to vaporize blood vessels to reduce the intake of substances that promote the growth of the scar [34]. The most common side effects are hyperpigmentation and hypopigmentation, followed by blister formation and postoperative purpura [35].

The intensity and severity of side effects depend on anatomy site, skin phototype, and racial differences.

Efficacy of laser treatment can be assessed with different techniques such as multispectral analysis, Raman spectroscopy, and high-resolution ultrasound.

Raman spectroscopy is a non-invasive technique that monitors the collagen presence on resurfaced skin and studies the collagen regeneration during the wound healing process of acne scars after laser procedures [36].

High-resolution ultrasound can be used to objectively measure the increase of dermal thickness and hydration, due to collagen neosynthesis and conformational changes in the extracellular matrix component, signs of the efficacy of the laser treatment; it can also predict efficacy by measuring the initial skin thickness [37].

The properties of multispectral analysis technique to hold the histological structure of skin disease and to investigate spectral features of abnormal parts, even if the normal parts have color variations, is an effective tool for capturing the information in a better way than other techniques.

Racial differences in skin pathophysiology have been reported based on genetic and environmental factors. The most frequent skin phototypes in the Asian population are Fitzpatrick types III or IV [38]. An essential difference between white and nonwhite skin is in the amount of epidermal melanin with larger melanosomes; no difference in melanocyte quantities is reported [39]. Melanosomes are degraded slower and absorb more laser energy in dark skin than white skin, which leads to damage to melanin cells resulting in an alteration of pigmentation (hypopigmentation, hyperpigmentation, and depigmentation) [40]. The thickness dermal, the more vulnerable melanocytes, and the increase in the reactivity of mesenchyme result in a higher risk of hypertrophic scars and keloid formation. As a result of acne, scarring and hyperpigmented macule are more common in darker skin [41].

Asian develop keloids and pigmentary problems more frequently than Caucasians, therefore, any procedures, especially laser treatment, must be carried out with caution [42].

In Asian skin, laser therapy can be used to treat various skin diseases such as vascular lesions, hypertrophic scars, keloids, striae, and pigment alteration [43].

The competition between laser energy and epidermal melanin makes lasers less effective.

The 585-nm PDL is the treatment of choice for hypertrophic scars and keloids on white skin, but the clinical improvement of thickness and viscoelasticity is lower in dark skin [44]. The treatment intervals and the appropriate energy should be selected with caution due to the risk of pigmentary alteration, which is higher than in fair-skinned patients [45].

Skin resurfacing to treat sun damage, wrinkles, and scars can be performed by CO_2_ laser [46]. PIH is the most common complication, especially in dark skin, developing during the first month and becoming more evident within four months [47].

Er: YAG laser is an alternative to the CO_2_ resurfacing lasers because of the lower depth of necrosis and the shorter thermal damage induced by Er: YAG laser, leading to lower melanocytic activity and minimizing the inflammatory reaction [48]. Postoperative complications are often less severe and resolve quicker than CO_2_ laser treatment [49]. Sometimes we can use combined CO_2_ and Er: YAG lasers to reduce the sequelae such as the post-laser erythema and induce a faster healing time and better cosmetic results [50]. We can perform a single-pass CO_2_ laser followed by Er: YAG laser to vaporize and reduce the thermal damage [51].

Fractional resurfacing systems utilize microscopical treatment zones of thermal injury to the skin, leading to some areas of untreated skin repopulating the damaged areas with faster recovery and fewer side effects [18]. The 755-nm Alexandrite picosecond laser improves acne scars in Asians [52].

The fractional picosecond laser is effective in the treatment of acne scars with minimal side effects [53]. Fractional handpieces focus high precision microbeams in a grid pattern to deliver the effect on the epidermis and, above all, on the dermis [54].

The Q-switched lasers have short impulses (nanoseconds or, even, picoseconds) with a power peak of mega- or giga-watts. Picosecond lasers differ from the nanosecond ones in that they deliver ultra-short pulse durations, which generate more photoacoustic effect and less photothermal damage. QSF Nd: YAG laser has already been used to treat post-surgical facial scars with decreased scar severity with mild and transient adverse events. To prevent and reduce the side effects of laser treatment in Asian skin, we use some regimens such as sun avoidance, sunscreen, and epidermal cooling [55].

## 5. Conclusions

Management of Asian skin requires different considerations because new laser technology, which prevents epidermal damages, is in high demand. This is the first study that confirms the efficacy and safety of QSF 1064 nm laser for scars treatment in Asian skin with excellent clinical results, little downtime, and no reported PIH. This study opens new horizons in the laser treatment of scars in Asians.

Limitations of this study include relatively small sample size, lack of a control group, and the absence of histopathologic assessments. Long-term follow-up on its efficacy and a larger sample size may be required in further studies to confirm our results. 

## Figures and Tables

**Table 1:** Goodman and Baron’s quantitative grading scale.

(Grade) Type	Number of lesions: 1 (1–10)	Number of lesions: 2 (11–20)	Number of lesions: 3 (21–30)
A Milder scarring (1 point each) B Moderate scarring (2 points each) C Severe scarring (3 points each) D Hyperplastic (papular) E Hyperplastic	1 point 2 points 3 points 2 points <5 cm^2^ Area 6 points	2 points 4 points 6 points 4 points 5–20 cm^2^ Area 12 points	3 points 6 points 9 points 6 points >20 cm^2^ Area 18 points

**Table 2:** Patients’ characteristics.

ID	Sex	Age	Photo Type	Acne Scarring Grading System before	Acne Scarring Grading System after 3 Months	Pain VAS	Side Effect
				treatment			
1	F	24	3	15	8	3	None
2	F	31	3	21	12	2	None
3	F	19	4	18	9	2	None
4	F	26	4	24	18	1	None
5	F	25	4	31	23	2	None
6	F	31	3	32	21	4	Erythema
7	F	42	2	13	4	1	None
8	F	28	4	28	15	2	None
9	F	29	3	37	26	3	None
10	F	20	3	25	13	2	None
11	F	33	4	21	9	1	None
12	F	37	4	17	6	2	None
13	F	29	3	30	18	2	None
14	M	19	3	21	16	3	Erythema
15	F	44	3	16	13	2	None
16	F	41	4	24	22	5	None
17	M	31	4	31	27	3	None
18	F	28	3	34	31	1	Erythema
19	F	32	4	35	16	5	None
20	F	37	4	14	14	2	None
21	F	21	4	10	4	4	None
22	F	26	4	18	11	3	None
23	F	24	3	21	9	2	None
24	F	21	3	13	5	3	None
25	F	27	3	15	9	2	None
26	F	18	4	24	12	2	None
27	F	37	4	21	11	1	None
28	F	34	3	17	9	3	None
29	F	46	3	29	19	3	None

## Data Availability

Data available form the corresponding author upon reasonable request.
